# Pea peels as a value-added food ingredient for snack crackers and dry soup

**DOI:** 10.1038/s41598-021-02202-5

**Published:** 2021-11-23

**Authors:** Mona M. H. Mousa, Mohammed A. El-Magd, Heba I. Ghamry, Mohammad Y. Alshahrani, Nora H. M. El-Wakeil, Eman M. Hammad, Galila A. H. Asker

**Affiliations:** 1 Food Science and Technology Department, Faculty of Home Economics, Al-Azhar University, Tanta, Egypt; 2 Anatomy Department, Faculty of Veterinary Medicine, Kafrelsheikh University, Kafrelsheikh, Egypt; 3 Department of Home Economics, College of Home Economics, King Khalid University, P.O. Box 9004, Abha, 61413 Saudi Arabia; 4 Research Center for Advanced Materials Science (RCAMS), King Khalid University, P.O. Box 9004, Abha, 61413 Saudi Arabia; 5 Department of Clinical Laboratory Sciences, College of Applied Medical Sciences, King Khalid University, P.O. Box 61413, Abha, 9088 Saudi Arabia; 6 Nutrition and Food Science Department, Faculty of Home Economics, Al-Azhar University, Tanta, Egypt

**Keywords:** Biological techniques, Ecology, Plant sciences

## Abstract

The food industry produces large quantities of waste, which is available in bulk at zero cost. This study aimed to investigate a new method to maximize the protein intake from pea peels and its further utilization as a value-added food ingredient to produce healthy snack crackers and dry soup. Dehydrated green curd of pea peel (DGCPp) with high protein content (35%) was prepared and incorporated into snack cracker and instant soup powder. Wheat flour was substituted with DGCPp to prepare crackers at three substitution levels (5, 10, and 15%) compared to the cracker control sample (100% wheat flour). Increasing the level of this substitution improved the nutritional value of crackers, with highest protein content was in DGCPp crackers (15%). Crackers also had higher contents of mineral and essential amino acids. The physicochemical and sensorial properties of soup samples were significantly influenced by the addition of DGCPp. Higher rehydration value and mineral content (Ca, Mg, Fe, and Zn) were observed in DGCPp soup samples compared to the control sample. Soup samples of all proportions were more acceptable by all the panelists compared with the control sample. With these findings, it can be concluded that DGCPp can be utilized in a variety of food products (such as crackers and soups) with higher nutritive values.

## Introduction

Peas (*Pisum sativum L*) are one of the most important vegetable crops grown around the world which have high protein (18–30%) content^1^. Peas can be grown in both frost-hardy and cold-climate environments. There are two types of peas: green peas marketed as fresh or canned, and dried yellow peas^2^. Pea wastes formed in great amounts during industrial processing cause significant environmental issues and can emit toxic gases^2,3^. Unsustainable waste disposal may also represent high economic costs since they have a direct impact on production profitability^4^. The pea peel wastes, which represent approximately 30–40% of the total pea weight, are available in bulk at zero cost^3^. Therefore, many approaches are required to convert these wastes into useful products with high nutritional value. Such approaches include reusing pea peel wastes as animal ration and using their bioactive compounds as a natural additive in food, cosmetics, and pharmaceutical applications.

Snack foods are globally widespread and utilized very well. Adults and children like to eat these foods, especially between regular meals. Crackers are thin, crisp wafers, commonly made of unsweetened and unleavened dough with yeast. Generally, crackers are manufactured with stronger flours than those used in cookie baking. Consumer demand for new functional snack foods with potential health benefits is increasing. Cereal proteins contain an inadequate amount of some essential amino acids, and because of the high cost of animal proteins, it becomes a mandatory demand to find out alternative sources of protein to support low price foods prepared from grains^5^. The instant dehydrated soup is very nutritional and can be used as an agent to provide protein in diets. It has a prolonged stable flavor that can last for approximately one year. Also, dry soups are easy to store at room temperature, easily prepared, available at all times, and lightweight for shipping. A balanced food can be achieved by incorporating whole cereals, vegetables, and dairy products. Such foods have a higher nutritive value and provide our bodies with all required calories^6^.

As animal protein deficiency is still a major issue in developing countries, alternative sources of protein are required. Plants are difficult to be utilized as protein sources due to their numerous non-digestible fiber fractions. By-products from cereals, legumes, and oil meals have been used by several researchers^7–9^ as a raw material for the recovery of proteins. Fruit and vegetable waste such as carrot peels, outer leaves of cabbage, potato peels, cauliflower by-products, tomato peel, and others has been used as a good source of polyphenols, dietary fibers, and antioxidants^10–13^. Pea pod waste was used for cellulolytic enzyme production, as a feed for goat bucks^14^, and ruminants^15^, and as a source of dietary fiber in biscuits^16^, bread^17,18^, cake ^17^ and instant soup^19^. Previous studies have shown that the extracted juice from pea peel is rich in protein and mineral contents^20,21^. However, the utilization of protein coagulate from pea peel juice in crackers and soup has not been investigated yet. The present study aimed to develop new cost-effective strategies for the valorization of the obtained protein from the pea peels to be used as an added food ingredient in snacks and soups using a thermal treatment for the green juice obtained after the separation of fibers.

## Results and discussion

### Chemical composition of wheat flour and DGCPp

The dried green curd of pea peel (DGCPp) had significantly higher contents of protein, ether extract, and ash on a dry weight basis than the wheat flour (Table 1). These results agreed with other studies^20,22–24^ who also found higher protein and mineral contents in the extracted juice from pea peel. DGCPp protein content (35.00 ± 2.28%) was higher than those of the pea pod powder (PPP, 11.99 ± 0.31%)^20^, which can be attributed to the new effective method used for DGCPp preparation in which insoluble dietary fiber and brown juice were eliminated to obtain a moist green curd with higher protein content. On the other hand, the ash content of DGCPp (8.12 ± 0.57%) was higher than that reported in PPP (4.61 ± 0.28%)^20^. Among all minerals detected, Ca (1080 ± 64.80 mg/100 g) was the highest which was also higher in DGCPp than wheat flour (Table 1). On similar lines, Abd-Allah et al.^21^ and El-Sharnouby^25^ found a similar Ca concentration (1037.99 mg/100 g) in PPP and (15.0 mg/100 g) in wheat flour. The contents of Na, Mg, Fe, and Zn were significantly higher in DGCPp than in wheat flour (Table 1). For their high mineral contents, DGCPp can be considered a good source of macro and micro elements and can be used in food fortification.

**Table 1:** Proximate chemical composition of wheat flour and DGCPp on a dry weight basis.

Components	Wheat flour	DGCPp
Moisture %	–	–
Crude protein %	15.25 ± 1.24 ^b^	35.00 ± 2.28 ^a^
Ether extract %	0.91 ± 0.05 ^b^	1.11 ± 0.07 ^a^
Ash %	0.61 ± 0.04 ^a^	8.12 ± 0.57 ^a^
Crude fiber %	0.70 ± 0.05	-
Total carbohydrates %	82.33 ± 5.09 ^a^	55.92 ± 4.62 ^b^
Available carbohydrates %	81.63 ± 7.40 ^a^	55.92 ± 4.62 ^b^
Ca (mg/100 g)	15.10 ± 0.91^b^	1080 ± 64.80 ^a^
Na (mg/100 g)	15.24 ± 0.91^b^	137.5 ± 8.25 ^a^
Mg (mg/100 g)	19.66 ± 1.18 ^b^	997.2 ± 49.86 ^a^
Fe (mg/100 g)	0.51 ± 0.04 ^b^	127.6 ± 7.65 ^a^
Zn (mg/100 g)	3.85 ± 0.26 ^b^	11.85 ± 0.83 ^a^
Energy value (kcal/100 g)	400.31 ± 32.50	372.23 ± 30.48

Data are expressed as mean ± SEM. Mean values with different superscript letters [a (the highest values)—b (the lowest value)] in the same row are significantly different at (*p* ≤ 0.05).

### Chemical composition of DGCPp crackers

The chemical analysis of crackers showed that the addition of DGCPp significantly increased protein and ash contents, with highest contents in 15% DGCPp crackers as compared to control (no DGCPp) crackers (Table 2). These results are in agreement with those reported by Abou El-Ez et al.^26^ who found that pea peel crackers were a higher source of protein and by Abd El-Salam et al.^27^ who found higher protein content of crackers following the addition of protein-rich algae. In contrast, moisture and crude fibers were significantly decreased in 15% DGCPp crackers relative to crackers prepared by 5 and 10% DGCPp. The decrement of moisture content could be due to the absence of fiber in DGCPp to hold more water during preparation. According to dietary references intakes DRI^28^, the daily intake of protein for adult 55 kg body weight of female and 65 kg body weight of male are 46 and 56 g protein per day. In the present study, each 100 g of 5%, 10%, and 15% DGCPp crackers covered 36.94, 40.61, and 45.91% for female and 30.34, 33.36, and 37.71% for male from daily intake of protein. DGCPp crackers had significantly higher content of Ca, Mg, Fe, and Zn, with highest concentrations in 15% DGCPp crackers than in control crackers (Table 2). This agrees with Garg^16^, who reported that the biscuits prepared from PPP are advantageous for people suffering from lifestyle diseases due to their high mineral content.

**Table 2:** Chemical composition of crackers prepared with DGCPp (g/100 g) on a dry weight basis.

Attribute	Control	Amount of DGCPp substitution (%)		
		5	10	15
Moisture %	9.18 ± 0.38^a^	9.87 ± 0.36^a^	9.74 ± 0.35^a^	8.76 ± 0.32^b^
Crude protein%	15.09 ± 0.51 ^c^	16.99 ± 0.55 ^b^	18.68 ± 0.60 ^b^	21.12 ± 0.83 ^a^
Ether extract %	4.51 ± 0.15	4.63 ± 0.14	4.40 ± 0.13	4.32 ± 0.11
Ash%	1.48 ± 0.04^c^	1.66 ± 0.04^b^	1.70 ± 0.05^b^	1.89 ± 0.06 ^a^
Crude fiber %	1.12 ± 0.05 ^a^	0.91 ± 0.04 ^b^	0.81 ± 0.03 ^c^	0.70 ± 0.03 ^d^
Total carbohydrates %	69.02 ± 4.65	65.95 ± 4.39	64.66 ± 4.11	63.21 ± 4.58
Available carbohydrates%	67.90 ± 6.83	65.04 ± 7.80	63.03 ± 6.93	62.51 ± 7.50
Ca (mg/100 g)	192.5 ± 13.42 ^c^	271.5 ± 15.13 ^b^	311.5 ± 16.03 ^a^	318.1 ± 15.68 ^a^
Na (mg/100 g)	2993.0 ± 102.33^a^	2183.5 ± 88.53 ^c^	2485.0 ± 94.01 ^b^	2608.5 ± 97.63 ^b^
Mg (mg/100 g)	390.0 ± 29.43 ^c^	475.5 ± 30.35 ^b^	553.0 ± 35.16 ^a^	572.5 ± 31.24 ^a^
Fe (mg/100 g)	2.60 ± 0.13 ^c^	8.85 ± 0.55 ^b^	15.10 ± 1.17 ^a^	16.65 ± 1.23 ^a^
Zn (mg/100 g)	9.05 ± 0.14 ^b^	9.68 ± 0.18 ^a^	9.73 ± 0.20 ^a^	9.95 ± 0.24 ^a^
Energy value (kcal/ 100 g)	375.43 ± 24.81	373.43 ± 44.81	372.96 ± 41.02	376.20 ± 45.14

Data are expressed as mean ± SEM. Mean values with different superscript letters [a (the highest values)—d (the lowest value)] in the same row are significantly different at (*p* ≤ 0.05). All groups were compared to each other.

### Amino acids content of DGCPp

DGCPp protein contained a lower content from threonine, valine, methionine, isoleucine, leucine, aromatic amino acids (phenylalanine and tyrosine), lysine, and histidine than required according to WHO/FAO/UNU^29^. Therefore, chemical score of these amino acids was lower than 100 (Table 3). On the other hand, the non-essential amino acids such as aspartic and glutamic existed in relatively high amounts. Amino acids are important indicators of protein quality and serve as the nitrogenous backbones for compounds like neurotransmitters and hormones. The essential amino acids are necessary for tissue maintenance and required for growth. Protein sources rich in glutamine have recently gained popularity because of this potential supporting the immune system and improving athletic performance^30^. Rowayshed et al.^31^ reported that the incorporation of available inexpensive by-products to foodstuffs, especially those deficient in amino acids, has a great economic value and a good standpoint in food technology and human nutrition. The biological value of protein is a measure of the efficient food protein and depends on how closely its amino acid reflects the amino acid pattern in the body tissues. The biological values were 100.09% in DGCPp. Protein-based food material has a good nutritional quality when its biological value reaches 70–100%.

**Table 3:** Amino acids profile of DGCPp compared with the requirement according to WHO/FAO/UNU.

Essential amino acid									
	Threonine	Valine	Methionine	Isoleucine	Leucine	Phenylalanine	Tyrosine^#^	Lysine	Histidine
DGCPp (mg/g)	5.42 ± 0.33	5.41 ± 0.27	1.72 ± 0.10	4.03 ± 0.24	8.17 ± 0.41	5.96 ± 0.36	2.91 ± 0.17	6.81 ± 0.34	6.49 ± 0.32
Requirement (mg/g protein)	23	39	16	30	59	38**		45	15
Chemical score	23.57 ± 1.18	13.87 ± 0.83	10.75 ± 0.65	13.43 ± 0.81	13.85 ± 0.69	23.34 ± 1.40**		15.13 ± 0.91	43.27 ± 2.16
Total			47.15 ± 2.36						
Biological value			100.09 ± 5.00						
Limiting score AA			10.75 ± 0.54 Methionine						
Non-Essential amino acid									
	Aspartic acid	Serine	Glutamic acid	Proline	Glycine	Alanine	Arginine		
DGCPp*	13.34 ± 0.80	4.69 ± 0.28	13.92 ± 0.69	5.20 ± 0.31	5.55 ± 0.38	4.78 ± 0.29	5.37 ± 0.32		
Total			52.85 ± 2.64						

*From WHO/FAO/UNU ^30^, **Phenylalanine + Tyrosine.

^#^Tyrosine is a non-essential amino acid, but it was mentioned within the essential amino acid for comparison with the permissible recommendations according to WHO/FAO/UNU ^30^ where the aromatic amino acids are mentioned together (phenylalanine and tyrosine).

### Physical properties of DGCPp crackers

**Figure 1:**
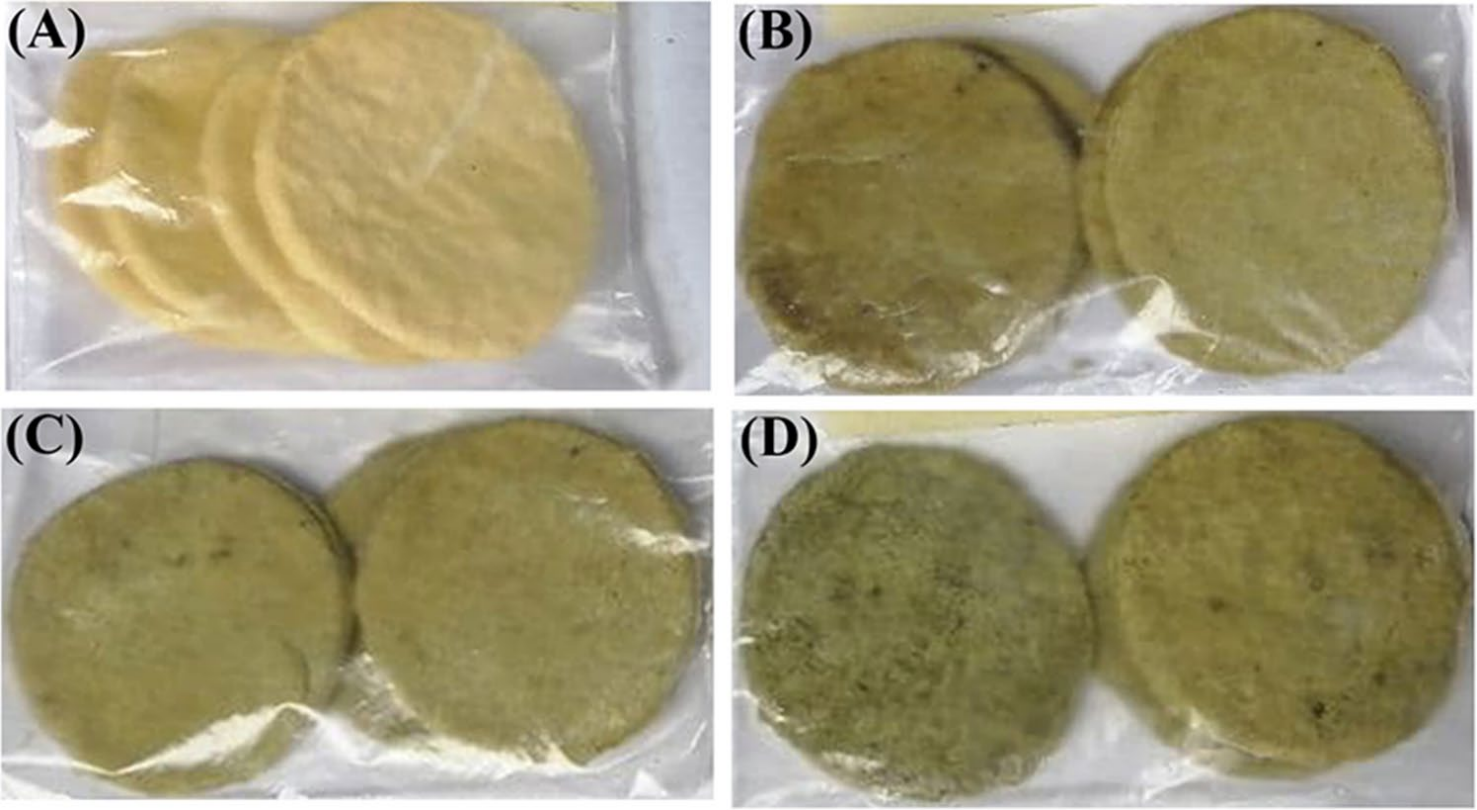
Crackers prepared by different % of DGCPp. (**A**), control; (**B**), 5% DGCPp; C, 10% DGCPp; D, 15% DGCPp.

DGCPp crackers had significantly lower diameters, with lowest diameters in 15% DGCPp crackers, than control crackers (Table 4, Fig. 1). The flour gluten could endure glass transition by heating, attaining flexibility that allows it to interact and form a network, boosting viscosity, and restricting the flow of crackers dough. The thickness and spread ratio properties are very crucial for the final shape of the crackers. With increasing DGCPp level, the thickness of crackers was significantly increased. The changes in diameter and thickness could affect the spread ratio ^32^. Consequently, we found that 15% DGCPp crackers showed the lowest spread ratio (Table 4). Crackers prepared with 15% DGCPp had a maximum weight relative to other crackers, whereas the control sample had a minimum weight. Similar results were obtained by Mihiranie et al.^33^, who found that the weights of all the snack crackers prepared by incorporating defatted coconut flour into wheat flour at 10, 20 and 30% (w/w) levels were higher than the weights of control crackers.

**Table 4:** Physical properties of crackers.

Blends	Diameter (mm)	Thickness (mm)	Spread ratio (D/T)	Weight (g)	Volume (cm^3^)	Specific volume V/W (cm^3^/g)
Control	31.79 ± 0.18 ^a^	2.07 ± 0.06 ^c^	13.56 ± 0.27 ^a^	1.50 ± 0.04 ^b^	3.60 ± 0.15	2.40 ± 0.09
5% DGCPp	30.92 ± 0.13 ^b^	2.59 ± 0.08 ^b^	11.86 ± 0.19 ^b^	1.55 ± 0.06^ab^	3.64 ± 0.12	2.35 ± 0.10
10% DGCPp	30.78 ± 0.19 ^cb^	2.67 ± 0.08 ^b^	11.04 ± 0.16 ^c^	1.65 ± 0.06 ^ab^	3.73 ± 0.13	2.24 ± 0.08
15% DGCPp	30.13 ± 0.15 ^c^	3.07 ± 0.09 ^a^	9.95 ± 0.18 ^d^	1.72 ± 0.05 ^a^	3.80 ± 0.12	2.21 ± 0.09

Data are expressed as mean ± SEM. Mean values with different superscript letters [a (the highest values)—c (the lowest value)] in the same column are significantly different at (*p* ≤ 0.05). All groups were compared to each other.

### Organoleptic evaluation of DGCPp crackers

Organoleptic evaluation is an important parameter to determine food quality. The 15% DGCPp crackers showed significantly lower values of color, taste, after taste, odor, general appearance, and overall acceptability than other crackers (Table 5). However, no significant difference was detected between 5 and 10% DGCPp and the control crackers. The crispness of the prepared sample with DGCPp was not affected compared to the control crackers. The score of overall acceptability ranged from 7.60 to 9.21. From these results we can conclude that the substitution of wheat flour with DGCPp could be up to the level of 15% in the cracker formulation. These results were consistent with Garg^16^ who found that in 30% pea pod powder biscuits, the overall acceptability declined while the taste and flavor of biscuits improved in 20% pea pod powder.

**Table 5:** Sensory quality attributes of crackers.

Attribute	Control	Amount of DGCPp substitution (%)		
		5	10	15
Color	9.43 ± 0.53 ^a^	8.82 ± 0.30^a^	8.51 ± 0.35^a^	6.92 ± 0.23^b^
Taste	9.33 ± 0.48 ^a^	9.04 ± 0.44 ^a^	8.83 ± 0.31 ^a^	7.61 ± 0.29^b^
Odour	8.81 ± 0.29 ^a^	8.83 ± 0.32^a^	8.74 ± 0.38^a^	7.83 ± 0.25^b^
Crispness	9.12 ± 0.64	9.10 ± 0.53	8.80 ± 0.52	8.55 ± 0.46
After taste	9.20 ± 0.35 ^a^	9.01 ± 0.33 ^a^	8.83 ± 0.38^a^	7.41 ± 0.18^b^
General appearance	9.22 ± 0.56 ^a^	8.93 ± 0.42 ^a^	8.55 ± 0.39^a^	7.13 ± 0.27^b^
Overall acceptability	9.21 ± 0.47^a^	8.81 ± 0.39 ^a^	8.78 ± 0.32^a^	7.60 ± 0.26^b^

Data are expressed as mean ± SEM. Mean values with different superscript letters [a (the highest values) and b (the lowest value)] in the same row are significantly different at (*p* ≤ 0.05). All groups were compared to each other.

### Physico-chemical properties of instant DGCPp soup powder

**Figure 2:**
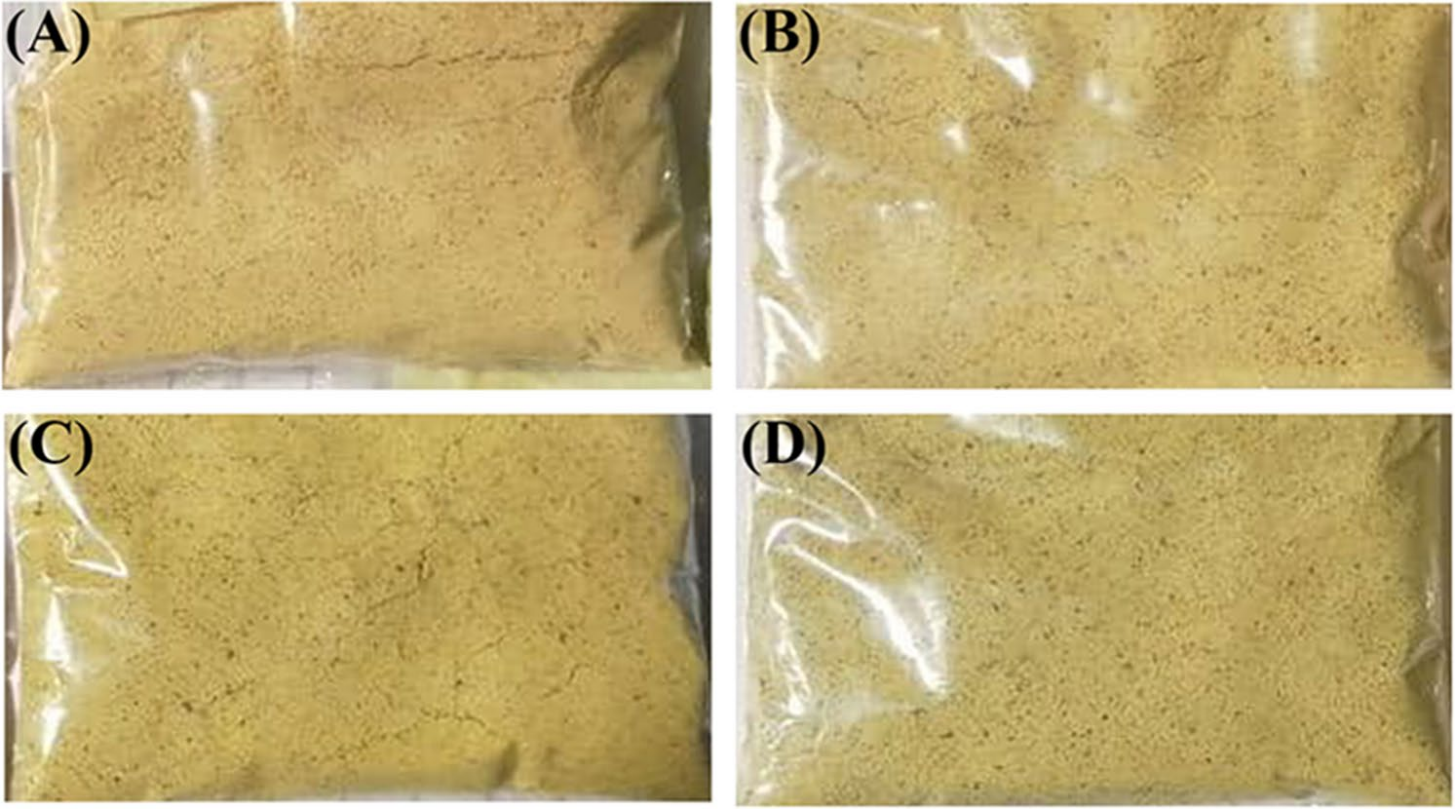
Dry soup prepared by different % of DGCPp. (**A**), control; (**B**), 5% DGCPp; C, 10% DGCPp; D, 15% DGCPp.

The physico-chemical properties of produced soup powder (Fig. 2) are presented in Table 6. Soup powder supplemented with DGCPp had significantly increased crude protein, ether extract, and ash, with highest levels in 15% DGCPp samples, as compared to the control samples. These results agreed with those of Abdel-Haleem and Omran^34^, who found increases in fat and protein content of the dried vegetarian soup mixtures following legumes supplementation. However, when DGCPp increased in the formulation, crude fiber, total carbohydrates, and available carbohydrates were significantly decreased in the soup powder.

**Table 6:** Physico-chemical properties of instant DGCPp soup powder.

Attribute	Control	Amount of DGCPp substitution (%)		
		5	10	15
Moisture %	9.42 ± 0.32	9.39 ± 0.41	9.36 ± 0.33	9.33 ± 0.38
Crude protein%	9.55 ± 0.23 ^d^	11.67 ± 0.47 ^c^	14.23 ± 0.53 ^b^	17.44 ± 0.62 ^a^
Ether extract %	3.12 ± 0.03 ^c^	3.26 ± 0.03 ^b^	3.30 ± 0.05 ^ab^	3.36 ± 0.05 ^a^
Ash%	6.67 ± 0.15 ^c^	6.96 ± 0.19 ^b^	7.36 ± 0.20 ^ab^	7.78 ± 0.23 ^a^
Crude fiber %	1.75 ± 0.04^a^	1.25 ± 0.03^b^	1.08 ± 0.02^c^	0.89 ± 0.02^d^
Total carbohydrates	69.44 ± 0.51^a^	67.40 ± 0.48^b^	64.67 ± 0.50 ^c^	61.20 ± 0.58^d^
Available carbohydrates	67.69 ± 0.50^a^	66.05 ± 0.82^b^	63.59 ± 0.63^c^	60.31 ± 0.73^d^
Energy value (kcal/ 100 g)	347.49 ± 21.28	344.07 ± 25.51	344.62 ± 21.53	342.80 ± 24.56
Ca	1049 ± 42.94^b^	1181.55 ± 40.57^a^	1183.10 ± 43.18^a^	1194.60 ± 42.73^a^
Na	3332.0 ± 123.24^a^	3100.0 ± 125.12^b^	3026.5 ± 111.59^b^	2919.0 ± 115.14^b^
Mg	691.5 ± 21.49^c^	664.0 ± 22.56^c^	764.5 ± 25.87^b^	900.5 ± 33.03^a^
Fe	3.10 ± 0.15^d^	4.35 ± 0.16^c^	8.50 ± 0.33^b^	14.95 ± 0.69^a^
Zn	16.95 ± 0.82^a^	11.55 ± 0.48^b^	11.2 ± 0.39^b^	10.9 ± 0.35^b^
Bulk density (g/ml)	0.79 ± 0.02	0.78 ± 0.04	0.76 ± 0.03	0.75 ± 0.04
Rehydration%	6.15 ± 0.15	6.16 ± 0.18	6.18 ± 0.12	6.22 ± 0.16
Dispersibility	74.46 ± 0.51 ^a^	71.89 ± 0.48 ^b^	71.25 ± 0.46 ^b^	69.42 ± 0.30 ^c^
Wettability (sec)	2.06 ± 0.06 ^c^	2.98 ± 0.11 ^b^	3.10 ± 0.08 ^b^	4.22 ± 0.10 ^a^
Water solubility index	56.67 ± 0.53 ^c^	63.67 ± 0.35 ^b^	65.67 ± 0.33 ^a^	66.33 ± 70.35 ^a^
Water activity	0.46 ± 0.02	0.46 ± 0.02	0.45 ± 0.01	0.45 ± 0.02
PH	5.50 ± 0.13 ^a^	5.12 ± 0.11 ^b^	4.97 ± 0.12 ^bc^	4.89 ± 0.09 ^c^

Data are expressed as mean ± SEM. Mean values with different superscript letters [a (the highest values)—d (the lowest value)] in the same row are significantly different at (*p* ≤ 0.05). All groups were compared to each other.

The recommended daily dose for adults from Ca, Na, Mg, Fe, and Zn were 750, 2092, 260, 17, and 15 mg, respectively. Ca, Mg, and Fe content of dried soup prepared by DGCPp supplementation was significantly higher than the control soup (Table 6). In contrast, Na and Zn contents were significantly lower in DGCPp soup than in the control soup. The calcium content of control soup sample provides 139.87% of the daily requirement (DRI) for adults, and substitute of wheat flour by DGCPp at 5, 10 and 15% in the soup formulation was shown to be more effective in covering the daily requirement of adults 140.21, 140.41, and 140.61%, respectively. Na content of the control, 5, 10 and 15% DGCPp soup could provide 159.27, 148.18, 144.67, and 139.53% from DRI for adults, respectively. Mg plays a vital role in energy production and nucleic acid synthesis^35,36^. Mg content of control sample and dried soup supplemented with different levels of DGCPp were 691.5 mg/100 g for the control sample and ranged between 664—900.5 mg/100 g for dried soup supplemented with 5,10 and 15% DGCPp. Fe is an important constituent of hemoglobin that has metabolic and enzymatic functions^37^. Dried soup supplemented with 5, 10 and 15% DGCPp provides 25.59,50 and 87.94% from DRI for adults. Overall, DGCPp can be recommended as a food supplement to help meet the recommended daily adult intake of some mineral elements.

Although particle size and initial moisture content of the flour are the main determinants of bulk density with greater bulk density when particles are smaller, we did not find a significant change in bulk density or rehydration among the four soups (Table 6). The dispersibility of the DGCPp supplemented soups was significantly lower than the control soup sample, suggesting weaker flour reconstitution in water for DGCPp soups^38^. Wettability determines the ability of flour to absorb water and inversely relates to time. The control sample had the lowest wetting time (2.06 s), while the highest wetting time (4.22 s) was recorded for dried soup supplemented with 15% DGCPp. The water solubility index of the DGCPp soups significantly increased, with highest value in 15% DGCPp, as compared to the control soup. The addition of DGCPp did not significantly change water activity (^a^w) values of the soup. However, all ranges (0.45–0.46) were promising as they are below those suitable for microbial growth (> 0.9)^39^. The pH of the control soup was significantly higher than all soups. These findings are consistent with Monteiro et al.^40^ who reported an inverse relation between pH and water solubility index and attributed this to the effect of temperature and water content.

### Viscosity of the instant DGCPp soups

**Figure 3:**
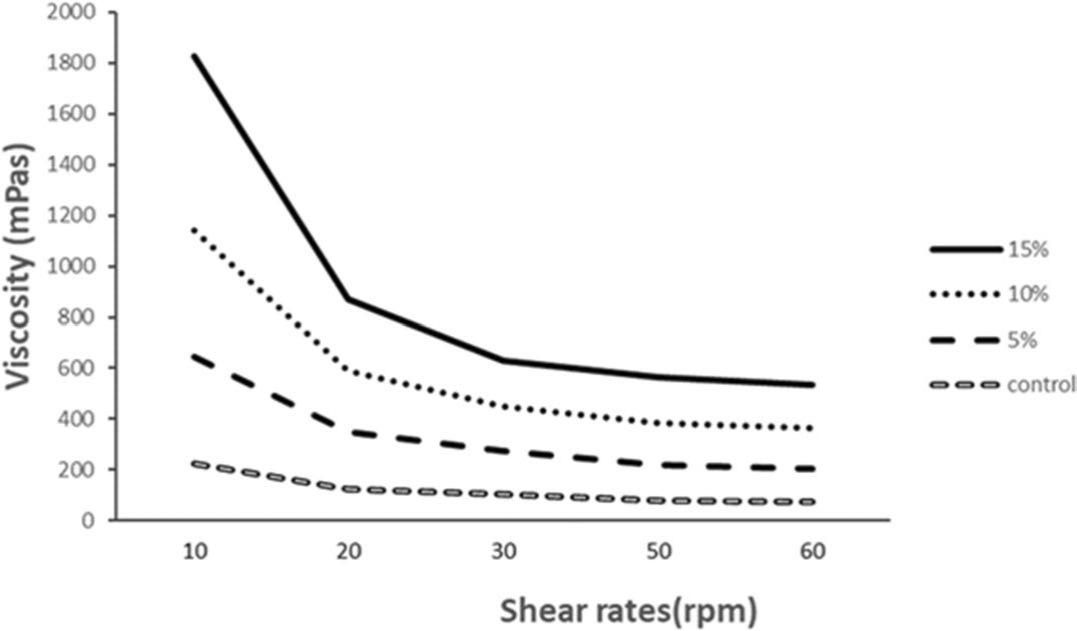
Viscosity of the resultant soup samples at different shear rates.

Soup viscosity of all soups reduced when the shear rate elevated (Fig. 3). This indicates that these soups are featured by a definite viscosity pattern that is probably recognized within the non-Newtonian pseudoplastic flow behavior. The 15% DGCPp soup recorded the highest viscosity pattern (685–170.13 mPas) as compared to the prepared soups by 10% (498.89–157.13 mPas), 5% (417.2–133.22mPas) DGCPp, and the control soup (226.29–73 mPas). The high viscosity of 15% DGCPp soup may be due to the greater percentage and the functionalities of DGCPp. These findings are consistent with Hanan et al.^41^ who reported a similar increase in the viscosity of soup pea pod where the peak viscosity of the control soups was 219.7 mPas. Soup samples prepared with DGCPp were more viscous. This increased viscosity could be due to the interaction between molecules, solubility in water which could be explained also due to inability of the soup powders to rehydrate and form crosslinks due to the high concentration of fiber which results in competition for the water absorption.

### Sensory evaluation of instant DGCPp soups

The addition of 15% DGCPp significantly decreased the taste, odor, appearance, color, thickness, and overall acceptability of the resultant soup samples as compared to the control sample (Table 7). DGCPp soup (10%) recorded the highest score of the quality attributes among the three DGCPp supplemented soups and was the most highly acceptable one. Similarly, Hanan et al.^41^ reported that when pea peel powder was increased above 12.5% in the soup formulation, the sensory score of soup decreased. Similarly, Belghith-Fendri et al.^42^ also found that elevation of pea pod powder above 15% in cake resulted in minimal sensory scores. We found that the moderate viscous DGCPp soup (10%) was the most acceptable one. In contrast, Hanan et al.^41^ and Ravindran, Matia-Merino^43^ found that high viscosity soup is more acceptable in the sensory evaluation than low viscous soup. Verma ^44^ also stated that psyllium husk in soup at higher percentages was found to have lower sensory scores for organoleptic characteristics.

**Table 7:** Sensory quality attributes of the instant DGCPp soup.

Attribute	Control	Amount of DGCPp Substitution (%)		
		5	10	15
Taste	9.12 ± 0.23^a^	9.01 ± 0.24^a^	9.21 ± 0.28^a^	8.20 ± 0.20^b^
Thickness	9.20 ± 0.19^a^	8.70 ± 0.13^b^	8.93 ± 0.14^ab^	8.62 ± 0.17^b^
Odor	9.43 ± 0.13 ^a^	8.82 ± 0.11 ^ab^	9.12 ± 0.14 ^a^	8.52 ± 0.10^b^
General appearance	9.70 ± 0.14 ^a^	9.02 ± 0.09^b^	9.04 ± 0.10^b^	8.61 ± 0.11^c^
Color	9.71 ± 0.12^a^	9.01 ± 0.08^b^	8.11 ± 0.12^c^	7.90 ± 0.12^c^
Overall acceptability	9.44 ± 0.15^a^	8.79 ± 0.14^bc^	8.90 ± 0.13^b^	8.41 ± 0.13^c^

Data are expressed as mean ± SD. Mean values with different superscript letters [a (the highest values)—c (the lowest value)] in the same row are significantly different at (*p* ≤ 0.05). All groups were compared to each other.

## Conclusions

The pea peels are available in bulk, at no cost and their disposal causes an environmental issue. This study showed the feasibility of producing a powder rich in protein and minerals from industry waste of green curd of pea peels (DGCPp) using a new, inexpensive method in which the green juice was first extracted from the pea peels, followed by heat coagulation of protein and separation of the coagulum by filtration and then its dryness. Through this new method, we can obtain moist green curd as a source of protein and the insoluble dietary fiber of pea peel as a source of cellulose. Also, the powder derived from these peels can be used to enhance the nutritive value in a variety of food products such as crackers and soups. Crackers containing 15% DGCPp had the highest nutritive value, while instant dehydrated soup containing 10% DGCPp was the most acceptable soup. Pea pods, which are only used for animal feeding, and if left, they would cause an environmental problem, and can be used as a new functional product to add value to various food applications**.**

## Materials and methods

The experimental research and collection of plant material comply with guidelines and legislation of Kafrelsheikh University.

### Materials

Green pea (*Pisum sativum*) peels were purchased from Kaha Company for canned food, Kaha city, Egypt. All-purpose wheat flour of extraction rate (72%), Plain (all-purpose), EL-Sabae shortening, sugar, sodium bicarbonate, fresh vegetables (tomato, carrots, potato, leek, onion, and garlic), skim milk powder, spices, salt (sodium chloride) were purchased from a local market, Kafr El-Sheikh City. All chemicals and solvents were obtained from Sigma-Aldrich chemical Co., Germany.

### Preparation of dehydrated green curd

**Figure 4:**
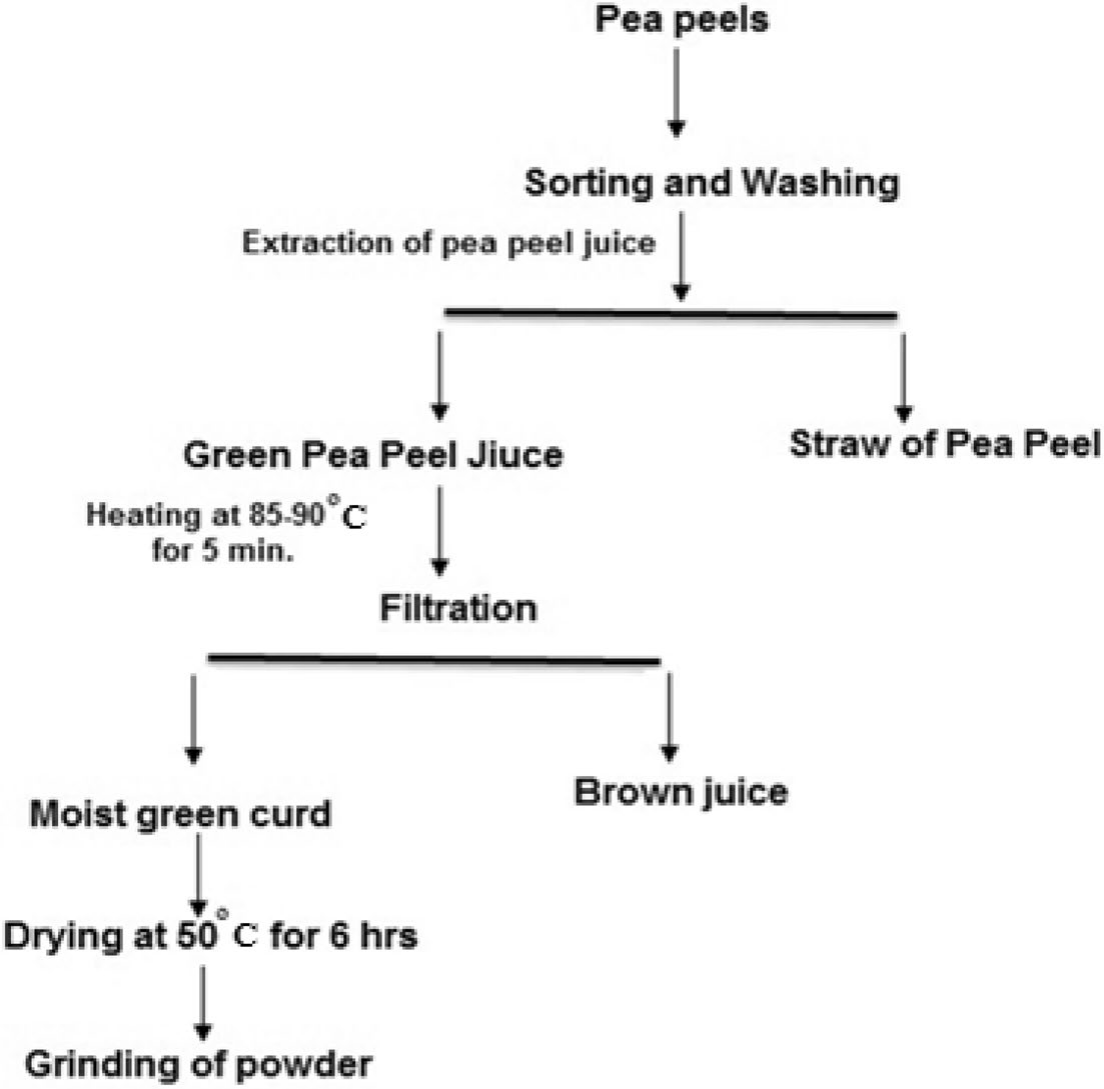
The flow chart of DGCPp processing.

The pea peels were sorted, washed, and kept in an open place at room temperature for two hours to remove moisture. The pea peels (1 kg) were put into a blender to extract pea peel juice (616.5 g) and insoluble dietary fiber (383.5 g). The juice was heated at 85–90 °C for 5 min to coagulate the protein. The coagulum (moist green curd 19.6%) was separated by vacuum filtration using a Büchner funnel fitted with the appropriate size filter paper and was dried in an electric oven (UNOX, XBC605, Italy) at 50 °C for 6 h to a final moisture content of 8.76%. Subsequently, the dried green curd of pea peels (DGCPp) was ground to a fine powder in an electric grinder (Model Moulinex type, No Y45, Spain), packed in low-density polythene bags, and stored in a cool and dry place for further uses (Fig. 4).

### Preparation of crackers formulations

Crackers were prepared as previously described^45^. In crackers recipe, the wheat flour (72%) was partially replaced by 5, 10, and 15% DGCPp (Table 8). Shortening, sugar, salt, and water were blended in a dough mixer using the flat beater for 1 min, then scraped down, and proceeded to blend at high speed for 3 min. Dry ingredients (soft wheat flour or its blends and baking powder) were gradually added to the mixture and blended for 3 min at slow speed. The resulting dough was left to rest for 5 min., and then sheeted by manual machine (Atlas 150 Wellness) to a thickness of 3 mm. using the templates with an outer diameter of 4 cm thickness; circle pieces of dough were formed. The crackers were baked at 150 °C for 25 min. Finally, crackers were allowed to cool at room temperature for 1 h. before sensory evaluations. The produced crackers were weighed and evaluated for physical and chemical attributes.

**Table 8:** Recipe formulation of crackers and instant DGCPp soup.

Ingredients	Crackers (g)				Ingredients	Instant DGCPp soup g/100 g			
	Control	Blend 1	Blend 2	Blend 3		Control	Blend 1	Blend 2	Blend 3
Wheat flour	100	95	90	85	Potato	40	40	40	40
Shortening	10	10	10	10	Milk Powder	25	20	15	10
Sugar	1	1	1	1	DGCPp	–	5	10	15
Salt	2	2	2	2	Carrot	15	15	15	15
Baking powder	3	3	3	3	Tomato	11	11	11	11
DGCPp	–	5	10	15	Onion	2	2	2	2
Water	58	58	58	58	Garlic	1	1	1	1

### Preparation of dehydrated soup mixtures

Fresh potatoes were washed, peeled, and cut into small cubes. The cubes were soaked in 0.2% potassium metabisulfite solution for 20 min, heat-dried at 40 °C for 18 h. until attaining 5% moisture^46^, milled, and sieved into a powder. A tomato was washed, sliced, and dried in an air oven dryer at 40 °C for 23 h. and powdered. Washed carrots and leek were cut into small pieces separately and blanched in boiling water for 2–3 min to inactivate enzymes and to get their natural color. Blanched vegetable pieces were chilled immediately in cold water, kept for few minutes to drain and dry at 40 °C for 23 h., and milled. Onion and garlic were peeled, washed and cut into slices. Sliced onion and garlic were dried at 40 °C for 18 h. and ground in an electric mill. The basic formulation of the dried soup mixture is presented in Table 8. Dried green curd of pea peel was used to substitute milk powder at 5, 10, and 15% concentration. Other ingredients were kept constant for all formulae. The ingredients were mixed well by a blender (according to each formula) then dried soup samples were packed separately in polyethylene bags and frozen at − 20 °C. Soup powder (25 g) was dissolved in 350 ml water, and the mixture was cooked for 5 min. and consumed in a way similar to the commercial soup powders.

### Proximate chemical composition

Moisture was measured by drying in an air oven at 105 °C. Crude protein was determined using the Micro-Kjeldahl method to determine the total nitrogen. The ether extract was estimated by a Soxhlet apparatus using the petroleum ether as a solvent. Ash content was determined by ashing in an Electric muffle at 550 °C until constant weight. Crude fibers were detected by digesting defatted sample with 1.25% H_2_SO_4_ flowed by 1.25% NaOH. Available carbohydrates were calculated by the following equation: available carbohydrates = total carbohydrate – crude fiber. The caloric value was calculated as follows: caloric value (k Cal/100 g) = (carbohydrates × 4) + (protein × 4) + (fat × 9). Minerals, calcium (Ca), sodium (Na), magnesium (Mg), iron (Fe), and zinc (Zn) were estimated using a pye Unicom SP 19,000 atomic absorption spectroscopy technology.

### Determination of DGCPp amino acids

Extraction of amino acids was performed using SYKAM S433 Amino Acids Analyzer as previously described^47^. After removal of fat, 5 mL 6 N HCl was added to 1 g of sample into the tube, tightly closed and incubated at 110 °C for 24 h. After incubation period, it was left for 10 min to cool, the solution was filtered and 200 μl of the filtrate was evaporated to dryness at 140 °C for 1 h. Each hydrolysate after dryness was diluted with 1 ml of sample dilution. After diluting the entire sample, it was filtered using a syringe filter 0.22 μm, in accordance with the amino acid standards (amino acid standards H, Pierce. Inc., Beckford). Aliquot of 150 μL of sample hydrolysate was injected in a cation separation column at 130 °C. after diluting it to 10% by sample dilution. The products of the reaction mixture were detected at wavelengths of 570 nm and 440 nm on a dual channel photometer. The amino acids composition was calculated from the areas of standards obtained from the integrator and expressed as percentages.

### The chemical score and biological value of DGCPp

Chemical score (CS)(%) = (EAA of crude protein / EAA of FAO/WHO) × 100^29^. Biological value was positively correlated with the lysine concentration and was calculated as follows: biological value (%) = 39.55 + 8.89 × lysine (g /100 g protein).

### Physical characteristics of crackers

Crackers weight (g), thickness (mm), diameter (mm), density (g/cm^3^), and spread ratio were measured as previously described ^48^. Six edge-to-edge crackers were used for evaluation and the average was recorded. Using a Vernier Caliper, diameter and thickness were determined. The spread ratio was calculated from the ratio of diameter to thickness. Specific volume was calculated as follows: specific volume = volume (ml)/ weight (g).

### Rheological properties of instant dehydrated soup samples

Rheological parameters (viscosity and shear rate) of dried soup samples were measured using Viscometer (Viscotech Myr VR 3000). The slurry was prepared by combining 10% of the soup mix with boiled water. The slurry was placed in a small sample beaker, spindle no. 2 selected for the measurement, and an embedded temperature sensor was used to maintain the desired temperature. The viscometer was operated between 10 and 60 rpm. Viscosity and shear rate data were obtained directly from the instrument. Rheological measurements were made for the resultant soup samples and controlled the temperature of viscometer at room temperature (25 °C ± 1 °C).

### Soup mixtures properties

The bulk density (mass/volume) and rehydration ratio (RR) were determined according to Chitomarat ^49^ and Krokida and Marinos-Kouris ^50^, respectively. The water solubility index was determined as previously described ^51^ and calculated by subtracting the weight of crucible after drying – weight of empty crucible × 100 / weight of the sample. The dispersibility was calculated as follows: % dispersibility = 100—the volume of the settled particle^51^. To determine the wettability of the flour samples, a glass funnel was put 10 cm above a beaker filled with 100 ml distilled water at 25 °C. The narrow opening of the funnel was closed by a tube and 1 g powder sample was added in the vicinity of the tube. The tube was elevated while the stopwatch was started simultaneously. Finally, the time for the powder to become fully wet was registered. Water activity was measured with a Rotronic (Hygrolab3, Switzerland). The ground sample (vegetable soup) was filled in the plastic cups to the top and the Hygroplam probe was inserted in these cups. After almost three to four minutes, the display showed the water activity reading, along with the temperature^52^. The pH value was determined by homogenizing 10 g of the soup powder with 100 ml distilled water for 30 s at room temperature using pH meter (JENCO 608, USA).

### Sensory evaluation

The cracker samples were organoleptically evaluated by ten panelists for their sensory characteristics. Cracker sample was served on white and odorless plates and water was provided for rinsing between samples. Panelists evaluated the tested cracker samples concerning color, taste, odor, crispness, after taste, general appearance, and overall acceptability attributes^53^. According to the method ^54^**,** dried soup samples were performed by 10 members of Food Science and Technology Department, Faculty of Home Economic, Al-Azhar University, Tanta. Twelve grams of dry soup mix were added to 150 ml of slightly hot water and stirred slowly until it boiled. After 5 min of boiling, an equal amount /volume of soup was served to the panelist with an evaluation form. Palatability tests were 6 items, taste (10), thickness (10), odor (10), general appearance (10), color (10), and overall acceptability (50) for soup samples were carried out.

### Statistical analysis

The statistical analysis was carried out using SPSS (version 11.0 SPSS Inc., Chicago, USA). The results were expressed as mean ± SEM. Data were subjected to variance analysis (ANOVA). Using Duncan's test at (*p* ≤ 0.05) the variations between means were tested for significance.

## Data Availability

The data presented in this study are available on request from the corresponding author.
